# MnO_2_ Cathodes Integrated with rGO for Highly Conductive Cement-Based Batteries

**DOI:** 10.3390/ma19214643

**Published:** 2026-10-31

**Authors:** Song Gu, Yongchun Xu, Yifeng Xia, Hao Zhang

**Affiliations:** 1 Zhaotong Expressway Investment and Development Co., Ltd., Zhaotong 657000, China; ztgszltzb@163.com (S.G.); ztsgtgsjgb@163.com (Y.X.); ztgsyyglb@163.com (Y.X.); 2 Key Laboratory of Advanced Civil Engineering Materials, School of Materials Science and Engineering, Tongji University, Shanghai 201804, China

**Keywords:** cement-based batteries, MnO_2_ cathode, reduced graphene oxide, electronic conductivity, structural energy storage

## Abstract

Cement-based batteries (CBBs) offer a new technical approach for constructing multifunctional structural energy storage systems that combine load-bearing and energy storage capabilities; however, the low intrinsic electronic conductivity of traditional cement-based composite cathodes severely limits their electrochemical performance. In this study, rGO was incorporated into the MnO_2_ cathode to construct a highly conductive MnO_2_@rGO composite cathode for Zn-MnO_2_ cement-based batteries. The interconnected rGO network forms continuous electron transport pathways between MnO_2_ particles while improving electrical contact between the active material and the current collector. Furthermore, the flake-like rGO framework promotes the uniform dispersion of MnO_2_ and mitigates particle agglomeration and structural degradation during repeated charge–discharge cycles. Thanks to these synergistic effects, the MnO_2_@rGO cathode achieved an electronic conductivity of 14.59 mS cm^−1^, representing a 6243.5% increase over pure MnO_2_. The assembled cement-based battery exhibited a discharge specific capacity of approximately 125 mAh g^−1^ at 0.01 mA and retained 80.7% of its initial capacity after 100 cycles. Electrochemical Impedance Spectroscopy (EIS) results showed that the charge transfer resistance was reduced from 148 Ω for pure MnO_2_ to 56 Ω, confirming accelerated interfacial charge transport and effective suppression of electrode polarization. This study reveals the crucial role of conductive carbon networks in overcoming the electronic transport bottleneck in cement-based electrodes and provides a scalable design strategy for developing highly conductive and multifunctional structured batteries.

## 1. Introduction

With the rapid development of renewable energy systems and distributed electronic devices, energy storage technologies that can be deeply integrated into the built environment have attracted widespread attention [1,2,3,4,5,6]. Traditional batteries are typically used as standalone functional units, and their integration into buildings and civil infrastructure often results in additional weight, bulk, and installation costs [7,8,9]. Cement-based batteries offer a new solution by transforming concrete or cement-based components from traditional passive load-bearing materials into multifunctional structural systems that combine mechanical support with electrochemical energy storage capabilities [10,11]. Given that cement-based materials offer advantages such as widely available raw materials, low cost, high mechanical stability, and compatibility with existing building fabrication processes, cement-based batteries show great application potential in areas such as smart buildings, self-powered sensors, emergency power supply, and large-scale structural energy storage [12,13,14]. However, the electrochemical performance of current cement-based batteries remains significantly inferior to that of traditional energy storage devices. This is primarily due to the complex structure of the cement matrix and its high overall resistance, which not only limits ion diffusion but also hinders electron transport and exacerbates interfacial polarization between the active electrode material and the surrounding electrolyte phase [15,16]. Therefore, improving charge transport kinetics while maintaining the mechanical integrity of the cement matrix is key to advancing cement-based batteries from proof-of-concept to practical structural energy storage applications [17,18,19].

Among the various cathode materials used in cement-based batteries, MnO_2_ has attracted widespread attention due to its high theoretical specific capacity, low cost, abundant availability, good environmental compatibility, and multiple variable oxidation states [20]. When paired with a zinc anode, MnO_2_ enables charge storage through reversible electrochemical reactions involving protons and Zn^2+^, making it suitable for aqueous cement-based energy storage systems [21,22]. However, MnO_2_’s low intrinsic electronic conductivity and slow reaction kinetics severely limit its actual active material utilization. These issues are further exacerbated when MnO_2_ is embedded in a cement matrix. Discontinuous electron transport pathways within cement-based composite electrodes and poor electrical contact between active particles lead to higher electrode polarization and charge transfer resistance [23]. Furthermore, MnO_2_ particles are prone to agglomeration during electrode preparation, which reduces their electrochemical active surface area and creates localized electrochemically inert regions. During repeated ion insertion and extraction cycles, MnO_2_ may also undergo structural distortion, particle cracking, and loss of electrical contact, ultimately leading to rapid capacity decay during cycling [24]. Therefore, establishing a continuous and stable conductive network for MnO_2_ while ensuring efficient ion transport is key to improving the utilization of active materials, accelerating interfacial charge transfer kinetics, and enhancing the cycling stability of MnO_2_-based cement batteries [25,26,27].

To address the charge-transport bottleneck of MnO_2_ cathodes, rGO is introduced as an effective conductive component [28,29,30]. Its two-dimensional flake structure, high electronic conductivity, large specific surface area, and good mechanical flexibility facilitate the formation of an interconnected electronic transport network within the electrode. When combined with MnO_2_, rGO can bridge isolated active particles, shorten the distance of electronic transport, and improve the electrical contact between MnO_2_ and the current collector. At the same time, the oxygen-containing functional groups remaining on the rGO surface serve as nucleation and anchoring sites for MnO_2_, thereby promoting the uniform dispersion of active particles and enhancing interfacial coupling within the composite [31,32,33]. Furthermore, the flexible rGO scaffold can cushion local structural deformations caused by repeated redox cycles and suppress the agglomeration, cracking, and electrical contact failure of MnO_2_ particles. These properties are particularly important for cement-based batteries, as the highly heterogeneous and high-impedance cement-based environment makes it more difficult to establish continuous conductive pathways compared to traditional coated electrodes [34,35]. Therefore, the composite of MnO_2_ and rGO is expected to simultaneously enhance electron transport capacity, accessibility of active sites, interfacial charge transfer kinetics, and structural stability, thereby providing an effective approach to overcoming the intrinsic electrochemical limitations of MnO_2_ cathodes in cement-based energy storage systems [36,37].

In this study, a MnO_2_@rGO composite cathode was developed for highly conductive Zn-MnO_2_ cement-based batteries. By introducing rGO, an interconnected conductive network was constructed within the cathode, while maintaining sufficient exposure of MnO_2_ active sites. Structural and chemical characterizations confirmed the successful integration of MnO_2_ with rGO, which facilitated efficient electron transport and improved interfacial stability. Compared with pristine MnO_2_, the optimized MnO_2_ @rGO electrode exhibited significantly enhanced electrical conductivity (14.59 mS cm^−1^) and reduced charge-transfer resistance. Benefiting from the improved electron transport and accelerated reaction kinetics, the MnO_2_@rGO cement-based battery delivered a higher discharge capacity of approximately 125 mAh g^−1^ and maintained 80.7% capacity retention after 100 cycles. Furthermore, the rGO network effectively suppressed MnO_2_ aggregation and preserved continuous conductive pathways during cycling, contributing to improved structural stability. These results demonstrate that rGO modification is an effective strategy to overcome the intrinsic conductivity limitation of MnO_2_ cathodes and provides a feasible approach for developing high-performance and durable cement-based energy storage systems.

## 2. Experimental Section

### Materials

Nickel foam (thickness: 1 mm, Guangjiayuan New Materials Co., Ltd., Kunshan City, China), zinc foil (thickness: 0.3 mm, Dingyuan Metal Products Co., Ltd., Shanghai, China), polyvinyl alcohol (1750 ± 50, Sinopharm Chemical Reagents Co., Ltd., Shanghai, China), manganese sulfate monohydrate (MnSO_4_·H_2_O, Sinopharm Chemical Reagents Co., Ltd., Shanghai, China), reduced graphene oxide (rGO, Suzhou Tanfeng Technology Co., Ltd., Suzhou, China), zinc oxide (ZnO, Sinopharm Chemical Reagents Co., Ltd., Shanghai, China), hydrochloric acid (HCl, Sinopharm Chemical Reagents Co., Ltd., Shanghai, China), ammonium persulfate ((NH_4_)_2_S_2_O_8_, Sinopharm Chemical Reagents Co., Ltd., Shanghai, China), carboxymethyl cellulose (CMC, Sinopharm Chemical Reagents Co., Ltd., Shanghai, China), styrene-butadiene rubber (SBR, Sinopharm Chemical Reagents Co., Ltd., Shanghai, China), Super P conductive carbon black (Sinopharm Chemical Reagents Co., Ltd., Shanghai, China), potassium hydroxide (KOH, 95%, General Reagents), lithium hydroxide (LiOH, Aladdin Reagents Co., Ltd., Shanghai, China), and P·II 52.5 cement (Jiangsu Xinning New Building Materials Co., Ltd., Nanjing, China).

## 3. Preparation

(a) Preparation of MnO_2_

First, 0.02 mol of manganese sulfate monohydrate (MnSO_4_·H_2_O) and 0.02 mol of ammonium persulfate ((NH_4_)_2_S_2_O_8_) were added to 100 mL of deionized water and stirred for 40 min. Subsequently, the resulting mixture was placed in an oil bath at 85 °C and allowed to react for 6 h. After the reaction, the product was filtered and washed several times with deionized water and anhydrous ethanol, respectively. Finally, the resulting product was dried in an oven at 80 °C for 12 h to obtain MnO_2_ powder.

(b) Preparation of the MnO_2_@rGO Cathode

MnO_2_, the binder, and Super P were mixed in a mass ratio of 8:1:1, where the binder consisted of CMC and SBR in a mass ratio of 6:4; an additional 5 wt% (by mass of MnO_2_) of rGO was added, and the mixture was formulated into a uniform slurry. Nickel foam was immersed in the slurry to obtain the target cathode with surface-loaded active material, which was then dried in an oven at 60 °C for 12 h.

(c) Preparation of the cement-based electrolyte

An excess of ZnO powder was added to a mixed solution containing 12 wt% KOH and 0.6 wt% LiOH. The mixture was magnetically stirred at 500 rpm at room temperature for 10 h, followed by standing for 1 day. The supernatant was collected to obtain a 12 wt% KOH-LiOH solution saturated with ZnO.

A 5 wt% polyvinyl alcohol (PVA) powder was added to deionized water and stirred magnetically at 500 rpm and 95 °C for 1 h until the PVA was completely dissolved. Stirring continued while cooling to room temperature. The previously prepared ZnO-saturated KOH-LiOH solution was slowly added to the PVA solution to obtain the PVA-KOH-LiOH(ZnO) composite solution.

During the preparation of the composite electrolyte, the water-to-cement ratio was fixed at 0.35, excluding the moisture introduced by the PVA–KOH–LiOH(ZnO) solution. The PVA–KOH–LiOH(ZnO) solution was thoroughly mixed with fresh cement, then poured into a mold and allowed to set for 1 day. After demolding, the specimens were placed in a standard constant-temperature and constant-humidity curing chamber and cured for 28 days.

(d) Battery Assembly

The prepared cement-based electrolyte slurry was poured into a cubic mold with a side length of 2 cm. A MnO_2_ cathode and a zinc anode were then inserted, with the distance between the two electrodes maintained at 2.0 ± 0.5 mm.

## 4. Characterization

X-ray diffraction (XRD, Bruker D8, Karlsruhe, Germany) was employed to analyze the crystallographic information of electrode materials. The diffraction patterns were collected over a 2θ range of 5–90° with a scanning rate of 2° min^−1^. Raman spectra were collected using a Raman spectrometer (Renishaw inVia, Wotton-under-Edge, UK) to investigate the structural characteristics, defect states, and carbon-related information of the electrode materials. The measurements were performed using a 532 nm laser excitation source within the wavenumber range of 100–2000 cm^−1^. Scanning electron microscopy (SEM, JSM 7800 F, Akishima, Japan) and high-resolution transmission electron microscopy (TEM, JEM-2100, Akishima, Japan) were utilized for morphological structure analysis. Elemental valence states were determined by X-ray photoelectron spectroscopy (XPS, Thermo Scientific K-Alpha, Waltham, MA, USA).

### Electrochemical Measurements

To ensure experimental consistency and accurate interpretation, electrochemical measurements were conducted at room temperature (25 °C). Cyclic voltammetry (CV) and electrochemical impedance spectroscopy (EIS) tests were performed using a CHI650D electrochemical workstation (CH Instruments, Inc., Austin, TX, USA). EIS measurements were conducted over a frequency range of 100 kHz–0.01 Hz with an AC amplitude of 5 mV. Charge–discharge testing was conducted using a Wuhan LAND CT2001A testing system (Wuhan LAND Electronic Co., Ltd., Wuhan, China).

For the ionic conductivity test, two identical perforated stainless-steel sheets were inserted into both sides of CSEs, and the EIS test was performed after molding and maintenance. The ionic conductivity was calculated according to Equation (1).

(1) $$\sigma =\frac{H}{S^{**}\cdot R_{b}}$$

where σ is the ionic conductivity of the cement-based electrolyte, H and S** are the thickness and the sectional area of the sample, respectively. The internal resistance of the electrolyte, R_b_, was calculated based on the intersection of the curves with the real part of the Nyquist plot with the real axis.

## 5. Result and Discussion

**Figure 1:**
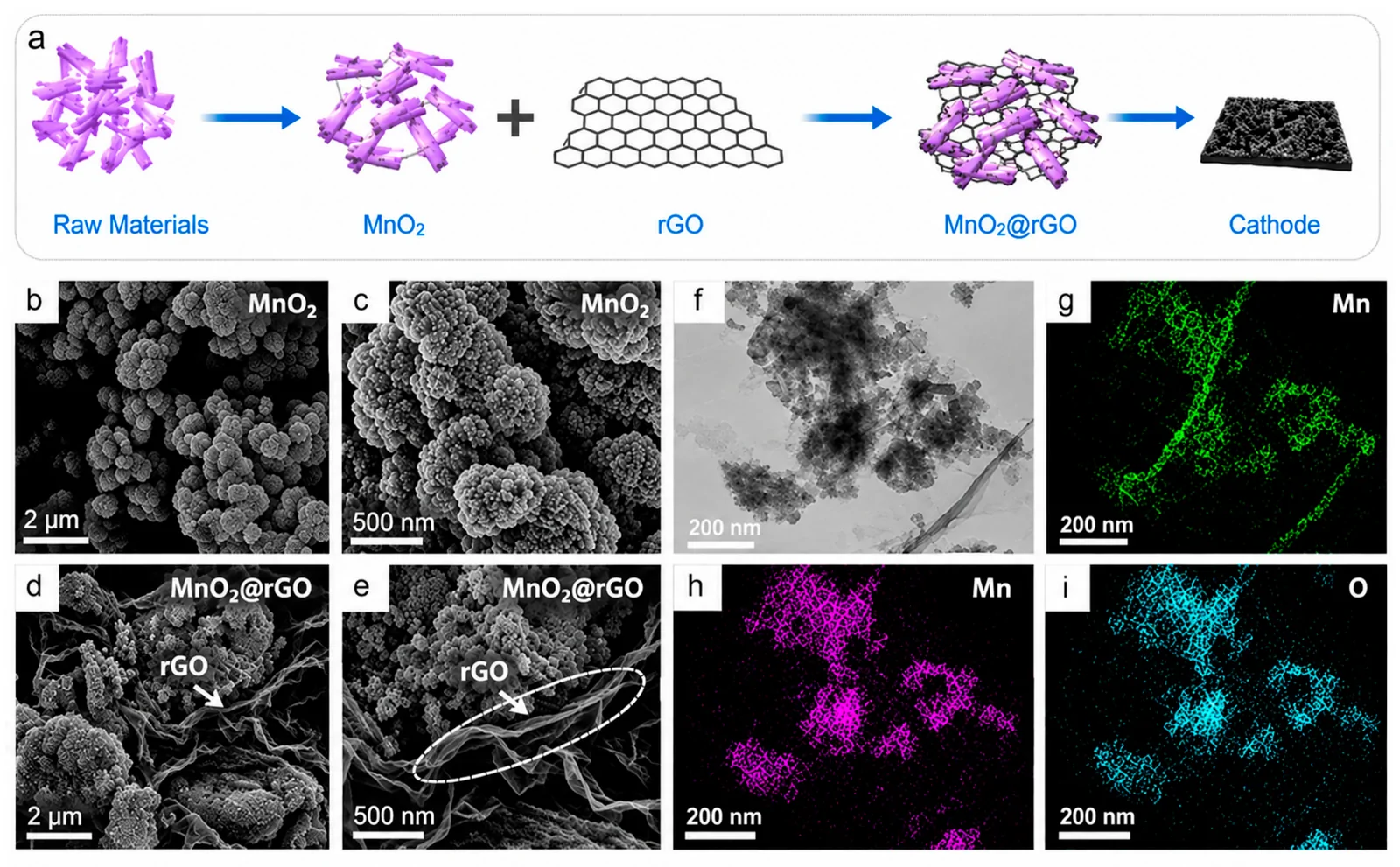
(**a**) Schematic illustration of the preparation process for the MnO_2_@rGO composite and its cathode; (**b**,**c**) SEM images of MnO_2_; (**d**,**e**) SEM images of MnO_2_@rGO; (**f**–**i**) mapping images of MnO_2_@rGO.

Figure 1 illustrates the preparation process and microstructural characteristics of the MnO_2_@rGO composite cathode. As shown in Figure 1a, MnO_2_ was first prepared from raw materials, then combined with rGO to obtain the MnO_2_@rGO composite, which was further processed into a cathode. The SEM images in Figure 1b,c show that pure MnO_2_ consists primarily of a large number of nanoscale particles aggregated into irregular micron-scale agglomerates; the particle surfaces are relatively rough, and the overall structure exhibits a distinct cluster-like arrangement. After the introduction of rGO, thin, wrinkled, flake-like structures can be clearly observed between the MnO_2_ particles (Figure 1d,e). These rGO layers are intercalated and distributed among the MnO_2_ agglomerates, forming relatively close contact with the surrounding particles, indicating that rGO has been successfully incorporated into the composite. Compared to pure MnO_2_, MnO_2_@rGO retains the basic morphology of MnO_2_ particles while incorporating a distinct two-dimensional layered structure. The TEM image in Figure 1f further shows that MnO_2_ particles are distributed on and around the surfaces of the translucent rGO layers, with close contact between MnO_2_ particles and rGO layers observable in localized areas. The corresponding elemental mapping results indicate that C, Mn, and O are all present in the selected regions. Among these, the distribution patterns of Mn and O are largely consistent, primarily concentrated in the MnO_2_ particle regions; C, on the other hand, is mainly distributed along the layered structure. The synergistic distribution of these three elements in the composite region further confirms the successful composite formation between MnO_2_ and rGO.

**Figure 2:**
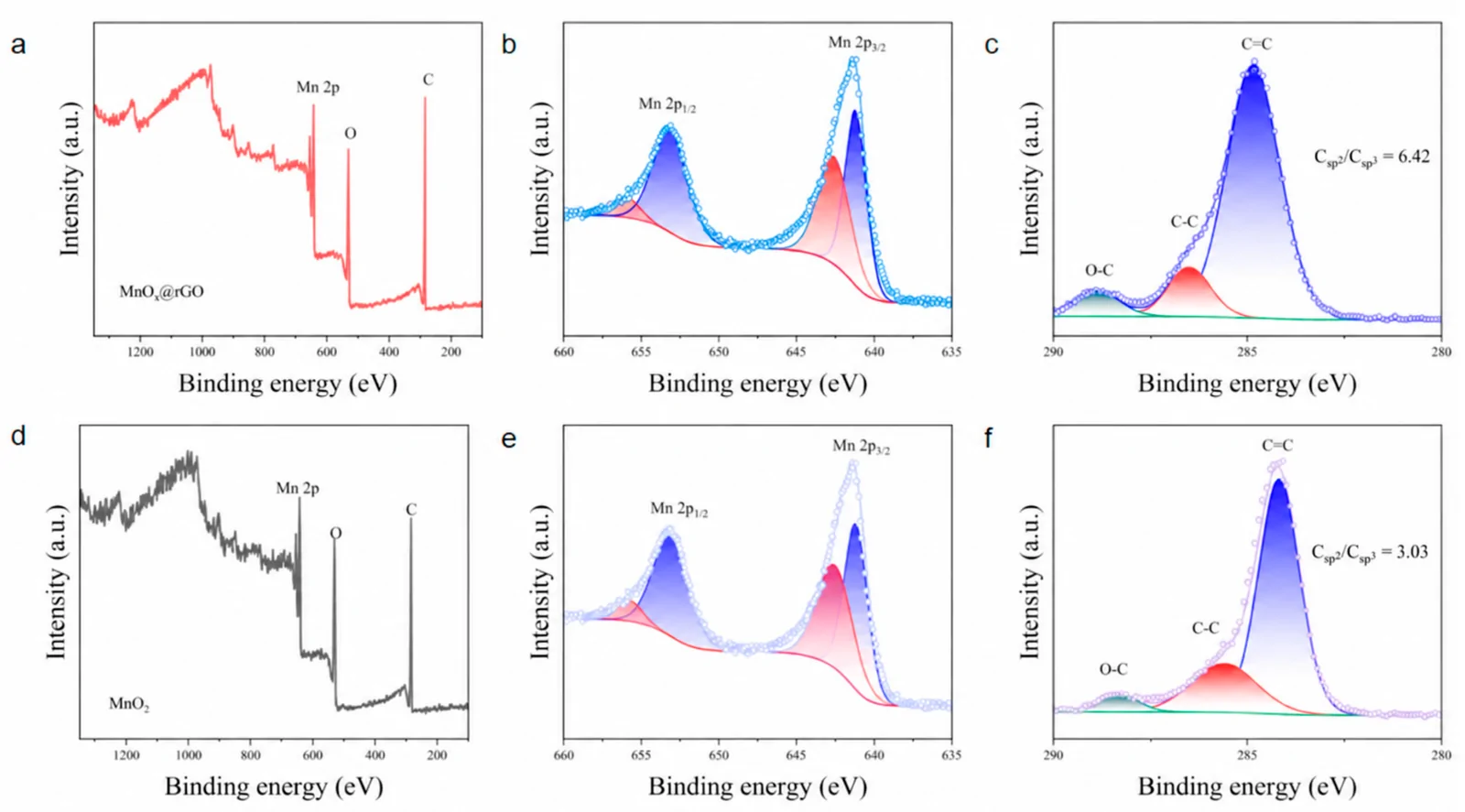
(**a**,**d**) Full XPS spectra of MnO_2_@rGO and MnO_2_; (**b**,**e**) high-resolution Mn 2p spectra; (**c**,**f**) high-resolution C 1s spectra.

Figure 2 shows the XPS analysis results for MnO_2_@rGO and pure MnO_2_, which were used to characterize the surface elemental composition and chemical state of the two materials. Distinct Mn 2p, O 1s, and C 1s characteristic signals can be observed in the full XPS spectrum of MnO_2_@rGO (Figure 2a) [38]. Compared with pure MnO_2_ (Figure 2d), MnO_2_@rGO exhibits a stronger C 1s signal, confirming that rGO has been successfully incorporated into the composite material. At the same time, distinct Mn and O signals are retained in both samples, indicating that MnO_2_ remains the primary active component after composite formation. Figure 2b,e show the high-resolution Mn 2p spectra of MnO_2_@rGO and MnO_2_, respectively. Both samples exhibit two characteristic peaks at approximately 642 and 654 eV, corresponding to Mn 2p_3/2_ and Mn 2p_1/2_, respectively. The peak positions and overall spectral profiles of the two materials are quite similar, indicating that the introduction of rGO did not significantly alter the primary chemical environment of the Mn element in MnO_2_. No new Mn-related characteristic peaks were observed after the composite formation, suggesting that MnO_2_ maintained good chemical stability during the preparation of MnO_2_@rGO. The high-resolution C 1s spectra shown in Figure 2c,f can be resolved into three components: C=C, C-C, and O-C. Among these, the C=C peak at approximately 284.8 eV corresponds to sp^2^-hybridized carbon, the C-C peak primarily corresponds to sp^3^-hybridized carbon, and the high-binding-energy O-C peak originates from residual oxygen-containing functional groups [39]. Compared to pure MnO_2_, the C=C peak in MnO_2_@rGO is significantly enhanced, and the C_sp_^2^/C_sp_^3^ ratio increases from 3.03 to 6.42, indicating a significant increase in the proportion of conjugated sp^2^ carbon structures in the composite. A higher proportion of sp^2^-hybridized carbon facilitates the formation of continuous electron transport pathways between MnO_2_ particles, while the residual oxygen-containing functional groups help enhance the interfacial contact between MnO_2_ and rGO. Overall, the XPS results confirm the successful incorporation of rGO, the basic preservation of the MnO_2_ chemical environment, and the significant increase in the proportion of conductive sp^2^ carbon in MnO_2_@rGO.

**Figure 3:**
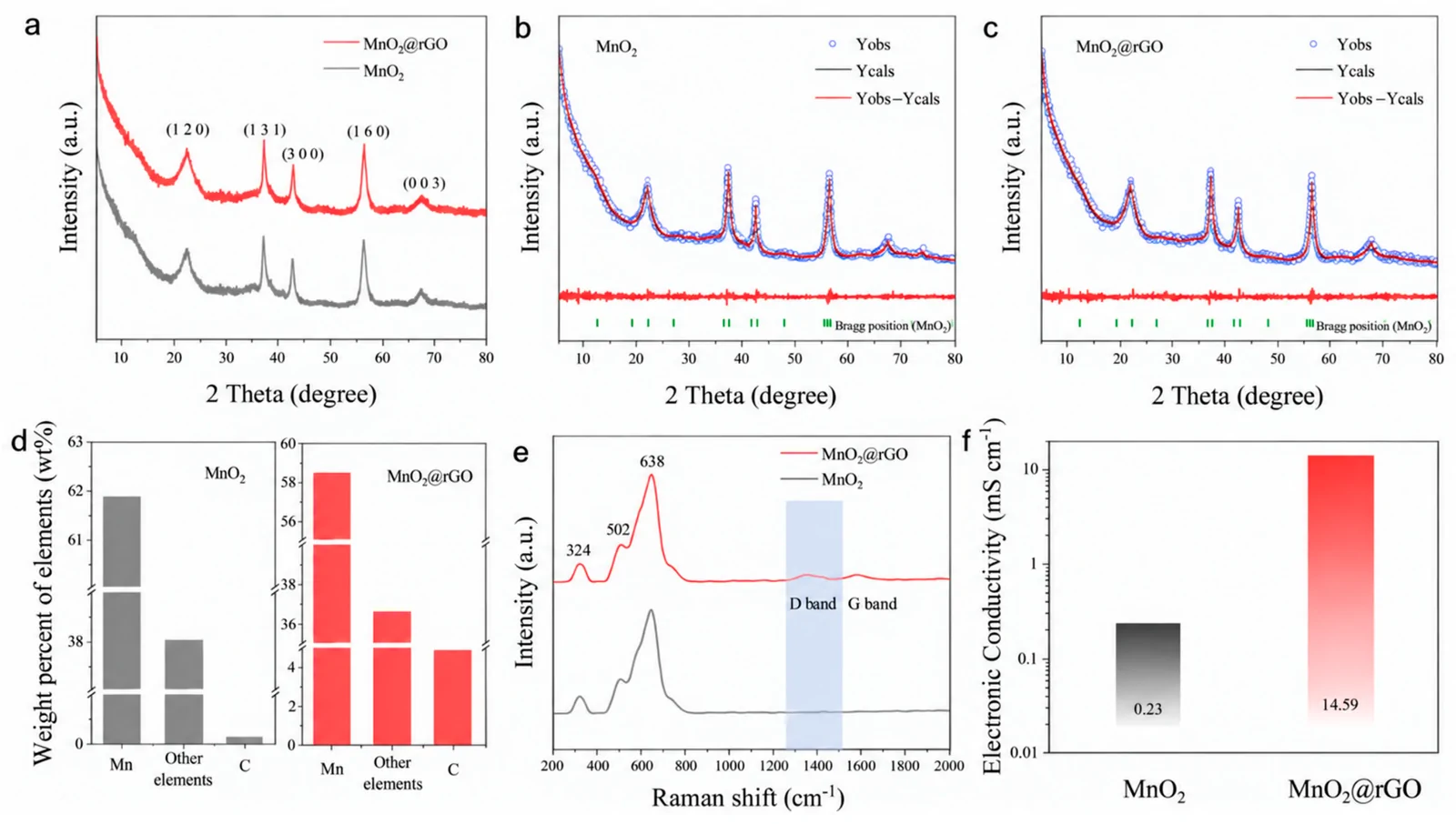
(**a**) XRD patterns of MnO_2_ and MnO_2_@rGO; (**b**,**c**) Rietveld refinement results; (**d**) elemental mass fractions; (**e**) Raman spectra; (**f**) electronic conductivity.

As shown in Figure 3a, both samples exhibit distinct diffraction peaks at approximately 22°, 37°, 42°, 56°, and 68°, corresponding to the (1 2 0), (1 3 1), (3 0 0), (1 6 0), and (0 0 3) crystal planes of MnO_2_, respectively. The diffraction peak positions of MnO_2_@rGO are essentially consistent with those of pure MnO_2_, and no obvious diffraction peaks from impurity phases were observed, indicating that the introduction of rGO did not significantly alter the main crystal structure of MnO_2_. The absence of distinct rGO-characteristic diffraction peaks in MnO_2_@rGO may be attributed to its low content and high degree of structural disorder. Figure 3b,c show the Rietveld refinement results for the two samples. The experimental diffraction patterns exhibit high consistency with the calculated curves, and the residual curves are generally smooth, further validating the phase composition of the samples. The elemental composition results are shown in Figure 3d. Pure MnO_2_ consists primarily of Mn and O, with small amounts of other elements; its carbon content is only about 0.2 wt%. Upon the introduction of rGO, the carbon content in MnO_2_@rGO increased to approximately 4.8 wt%, while the mass fraction of Mn correspondingly decreased, confirming the successful incorporation of rGO into the composite material. Figure 3e shows the Raman spectra; both samples exhibit characteristic vibrational peaks of MnO_2_ at approximately 324, 502, and 638 cm^−1^. Compared with pure MnO_2_, MnO_2_@rGO further exhibits distinct D and G bands at approximately 1350 and 1590 cm^−1^, which are associated with defect structures and the sp^2^-hybridized carbon framework in the carbon material, respectively, further confirming the successful composite formation of MnO_2_ and rGO [40]. Electrical conductivity measurements showed that the electrical conductivity of pure MnO_2_ was only 0.23 mS cm^−1^, whereas that of MnO_2_@rGO increased to 14.59 mS cm^−1^, approximately 63.4 times that of pure MnO_2_ (Figure 3f). These results indicate that the introduction of rGO significantly improved the electronic transport properties of the MnO_2_ composite [41].

**Figure 4:**
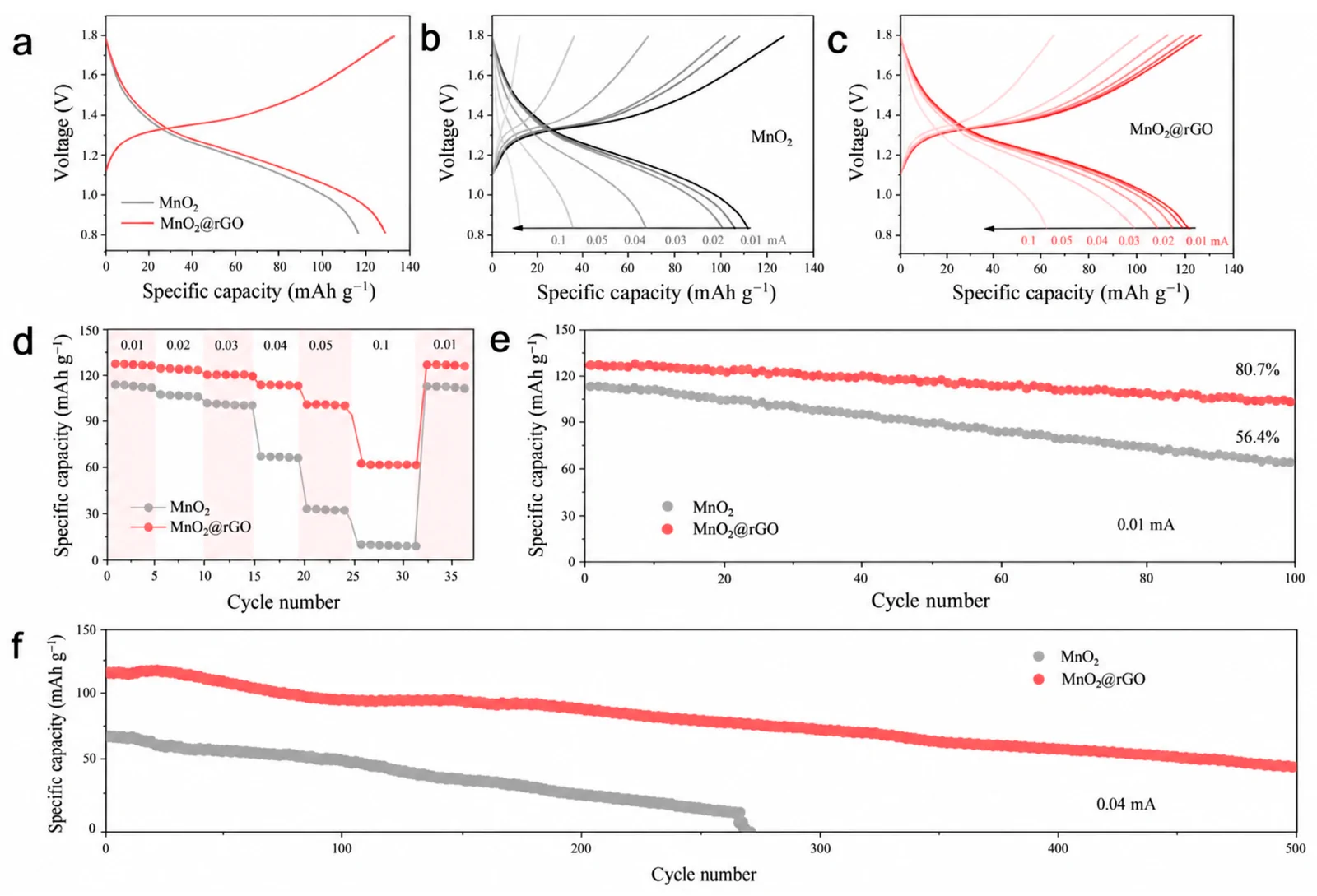
(**a**) Initial constant-current charge–discharge curve at 0.01 mA; (**b**,**c**) charge–discharge curves of MnO_2_ and MnO_2_@rGO cement-based batteries at different currents; (**d**) rate performance at 0.01–0.1 mA; (**e**) cycling performance after 100 cycles at 0.01 mA; (**f**) long-term cycling performance after 500 cycles at 0.04 mA.

As shown in Figure 4a, at 0.01 mA, the first-cycle specific capacity of the MnO_2_@rGO battery was approximately 125 mAh g^−1^, which was higher than the approximately 115 mAh g^−1^ of the MnO_2_ battery, and it also exhibited a longer charge–discharge curve. Figure 4b,c show the charge–discharge curves of the two batteries at currents of 0.01, 0.02, 0.03, 0.04, 0.05, and 0.1 mA, respectively. As the current increases, the discharge specific capacity of both batteries gradually decreases, and the voltage difference between the charge and discharge curves correspondingly increases; however, the MnO_2_@rGO battery maintains a higher capacity and a more stable voltage response at all tested currents. Figure 4d further compares the rate performance of the two batteries. At currents of 0.01, 0.02, 0.03, 0.04, 0.05, and 0.1 mA, the specific discharge capacity of MnO_2_@rGO was approximately 125, 120, 115, 105, 95, and 63 mAh g^−1^, respectively, all of which were significantly higher than those of the pure MnO_2_ battery. When the current was restored to 0.01 mA, the capacity of MnO_2_@rGO recovered to approximately 123 mAh g^−1^, close to its initial level, indicating good rate reversibility. Figure 4e shows that after 100 cycles at 0.01 mA, MnO_2_@rGO still retained a specific discharge capacity of approximately 104 mAh g^−1^, corresponding to a capacity retention rate of 80.7%; in contrast, MnO_2_ retained only about 65 mAh g^−1^, with a capacity retention rate of 56.4%. At a higher current of 0.04 mA, MnO_2_@rGO still exhibited a discharge specific capacity of approximately 46 mAh g^−1^ after 500 cycles, whereas the capacity of MnO_2_ continued to decline rapidly, approaching zero after approximately 270 cycles (Figure 4f). These results demonstrate that the incorporation of rGO significantly enhances the capacity output, rate performance, and long-term cycling stability of the MnO_2_ cathode.

**Figure 5:**
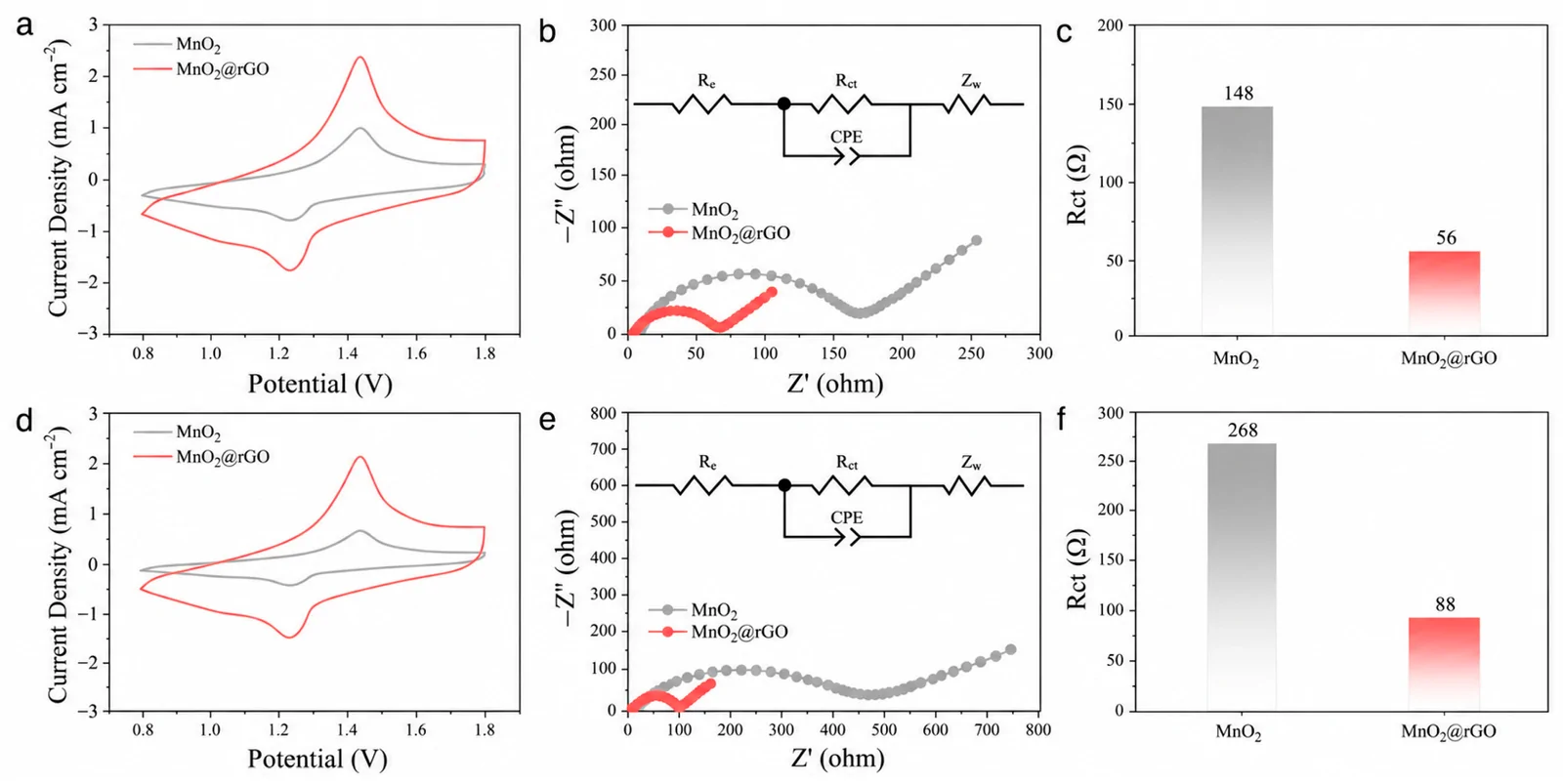
(**a**,**d**) CV curves of MnO_2_ and MnO_2_@rGO before cycling and after 200 cycles; (**b**,**e**) Nyquist plots and corresponding equivalent circuits, where R_s_ represents the series resistance, R_ct_ represents the charge-transfer resistance, CPE represents the constant phase element, and Z_w_ represents the Warburg impedance; (**c**,**f**) fitted charge-transfer resistance (Rct) values.

As shown in Figure 5a, both cement-based batteries exhibited distinct redox responses, indicating that reversible electrochemical reactions can occur at the MnO_2_ cathode. Compared with the pure MnO_2_ battery, the MnO_2_@rGO battery exhibited higher redox peak currents and a larger area under the CV curve, suggesting higher electrochemical activity and more efficient utilization of the active material. At the same time, the oxidation and reduction peaks of the MnO_2_@rGO battery are more distinct, suggesting that the introduction of rGO improves the reversibility of electrode reactions and reduces polarization during charging and discharging. Figure 5b shows the Nyquist plots of both batteries before cycling; the spectra consist primarily of a semicircle in the high-frequency region and a sloping line in the low-frequency region, corresponding to interfacial charge transfer and ion diffusion processes, respectively. The MnO_2_@rGO battery exhibits a significantly smaller semicircle diameter in the high-frequency region, indicating lower interfacial charge transfer resistance. Based on fitting using the equivalent circuit shown in the inset, the charge transfer resistance (Rct) for MnO_2_ and MnO_2_@rGO is 148 and 56 Ω, respectively (Figure 5c). Compared to pure MnO_2_, the Rct of MnO_2_@rGO was reduced by approximately 62.2%, indicating that the conductive network formed by rGO effectively improves electronic contact between particles and accelerates interfacial charge transfer. After 200 cycles, both the peak current and the integrated area of the CV curves for both cells decreased, indicating a certain degree of decline in electrode reactivity during long-term cycling (Figure 5d). However, the MnO_2_@rGO cell still retained a distinct redox peak, with its peak current and CV area remaining significantly higher than those of the pure MnO_2_ cell, indicating that the composite cathode still exhibits good electrochemical reversibility after long-term cycling. The post-cycling Nyquist plots showed that the semicircles in the high-frequency region of both cells had increased (Figure 5e), indicating that the electrode/electrolyte interface impedance gradually increased with cycling. Fitting results show that after 200 cycles, the Rct values for MnO_2_ and MnO_2_@rGO increased to 268 and 88 Ω, respectively (Figure 5f). Notably, the Rct of MnO_2_@rGO remained approximately 67.2% lower than that of pure MnO_2_. Furthermore, the Rct of pure MnO_2_ increased by approximately 81.1% compared to its initial value, whereas that of MnO_2_@rGO increased by only about 57.1%, suggesting that rGO not only reduces the initial interfacial impedance but also slows the increase in charge transfer resistance during cycling. These results indicate that the MnO_2_@rGO electrode exhibits faster interfacial charge transfer, lower reaction polarization, and more stable long-term electrochemical kinetics.

**Figure 6:**
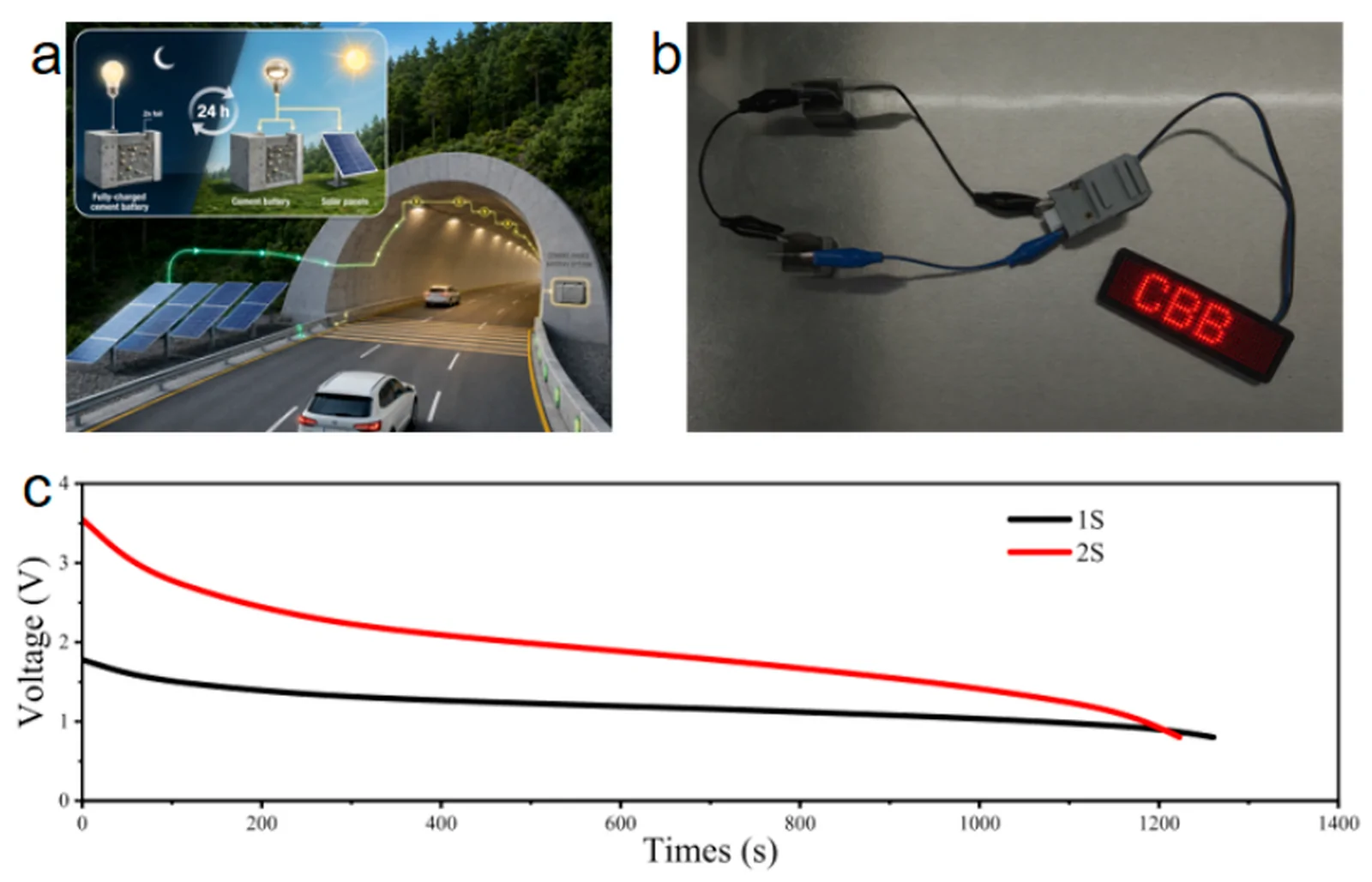
(**a**) Schematic diagram illustrating the application of a cement-based battery integrated with a solar power system for continuous tunnel lighting; (**b**) photograph of an LED display powered by a cement-based battery; (**c**) discharge curves for a single-cell battery (1S) and two cells connected in series (2S).

As shown in Figure 6a, this battery can be integrated with a photovoltaic system to store solar energy during the day and power tunnel lighting at night, which effectively addresses the intermittency defect of photovoltaic power generation and endows this structural energy storage device prominent engineering and economic feasibility for large-scale photovoltaic-tunnel lighting integrated projects. Figure 6b shows that the assembled battery successfully powers an Light Emitting Diode (LED) display, demonstrating its practical power supply capability. The discharge curves in Figure 6c show that the initial voltage of a single cell is approximately 1.8 V, while the initial voltage increases to about 3.6 V when two cells are connected in series. The series-connected cells maintain a higher output voltage during discharge, indicating that the operating voltage can be effectively adjusted by connecting cells in series.

The enhanced electrochemical performance of MnO_2_@rGO can be attributed to the synergistic effect between MnO_2_ active materials and the rGO conductive framework. The structural and chemical characterizations confirm the successful integration of rGO with MnO_2_, while the increased electronic conductivity demonstrates the formation of efficient electron transport pathways. During battery operation, the rGO network facilitates rapid electron transfer and reduces electrode polarization, thereby improving MnO_2_ utilization and stabilizing the electrochemical reaction process. As a result, the MnO_2_@rGO cathode delivers enhanced capacity retention and cycling stability compared with pristine MnO_2_, demonstrating the effectiveness of conductive network engineering for high-performance cement-based batteries.

## 6. Conclusions

In this study, a highly conductive MnO_2_@rGO cathode for cement-based batteries was fabricated by compositing MnO_2_ with an interconnecting rGO network. Structural and compositional characterization confirmed that rGO was successfully incorporated into the composite without significantly altering the primary crystal structure or chemical state of MnO_2_. The incorporation of rGO increased the electrode’s electronic conductivity from 0.23 mS cm^−1^ to 14.59 mS cm^−1^ and reduced the initial charge transfer resistance from 148 Ω to 56 Ω. Consequently, the MnO_2_@rGO cement-based battery exhibited higher discharge specific capacity, better rate performance, and more stable cycling performance. After 100 cycles at 0.01 mA, the capacity retention rate reached 80.7%, which is higher than the 56.4% observed for the pure MnO_2_ battery. Furthermore, after 500 cycles at 0.04 mA, the MnO_2_@rGO battery still maintained a discharge specific capacity of approximately 46 mAh g^−1^ and retained a relatively low charge transfer resistance after cycling. The assembled batteries can also have their output voltage increased by connecting them in series, successfully powering an LED display. These results indicate that constructing a continuous rGO conductive network effectively alleviates the electron transport limitations of the MnO_2_ cathode and enhances the practical electrochemical performance of cement-based batteries.

## Data Availability

The original contributions presented in this study are included in the article. Further inquiries can be directed to the corresponding author.
